# 736 Burn Injuries During the Pandemic: Older Patients, Delays to Admission, and Longer Hospital Stays

**DOI:** 10.1093/jbcr/irad045.211

**Published:** 2023-05-15

**Authors:** Jonathan Vuillier, Siam Rezwan, Rena Atayeva, Carisa Cooney, Emily Werthman, Rafael Felix Tiongco, Jessica Ballou, Julie Caffrey

**Affiliations:** Johns Hopkins University School of Medicine, Baltimore, Maryland; Johns Hopkins University School of Medicine, Baltimore, Maryland; Johns Hopkins University School of Medicine, Baltimore, Maryland; Johns Hopkins University School of Medicine, Baltimore, Maryland; Johns Hopkins Bayview Medical Center, Baltimore, Maryland; Johns Hopkins University School of Medicine, Baltimore, Maryland; Johns Hopkins University School of Medicine, Baltimore, Maryland; Johns Hopkins University School of Medicine, Baltimore, Maryland; Johns Hopkins University School of Medicine, Baltimore, Maryland; Johns Hopkins University School of Medicine, Baltimore, Maryland; Johns Hopkins University School of Medicine, Baltimore, Maryland; Johns Hopkins University School of Medicine, Baltimore, Maryland; Johns Hopkins Bayview Medical Center, Baltimore, Maryland; Johns Hopkins University School of Medicine, Baltimore, Maryland; Johns Hopkins University School of Medicine, Baltimore, Maryland; Johns Hopkins University School of Medicine, Baltimore, Maryland; Johns Hopkins University School of Medicine, Baltimore, Maryland; Johns Hopkins University School of Medicine, Baltimore, Maryland; Johns Hopkins University School of Medicine, Baltimore, Maryland; Johns Hopkins University School of Medicine, Baltimore, Maryland; Johns Hopkins Bayview Medical Center, Baltimore, Maryland; Johns Hopkins University School of Medicine, Baltimore, Maryland; Johns Hopkins University School of Medicine, Baltimore, Maryland; Johns Hopkins University School of Medicine, Baltimore, Maryland; Johns Hopkins University School of Medicine, Baltimore, Maryland; Johns Hopkins University School of Medicine, Baltimore, Maryland; Johns Hopkins University School of Medicine, Baltimore, Maryland; Johns Hopkins University School of Medicine, Baltimore, Maryland; Johns Hopkins Bayview Medical Center, Baltimore, Maryland; Johns Hopkins University School of Medicine, Baltimore, Maryland; Johns Hopkins University School of Medicine, Baltimore, Maryland; Johns Hopkins University School of Medicine, Baltimore, Maryland; Johns Hopkins University School of Medicine, Baltimore, Maryland; Johns Hopkins University School of Medicine, Baltimore, Maryland; Johns Hopkins University School of Medicine, Baltimore, Maryland; Johns Hopkins University School of Medicine, Baltimore, Maryland; Johns Hopkins Bayview Medical Center, Baltimore, Maryland; Johns Hopkins University School of Medicine, Baltimore, Maryland; Johns Hopkins University School of Medicine, Baltimore, Maryland; Johns Hopkins University School of Medicine, Baltimore, Maryland; Johns Hopkins University School of Medicine, Baltimore, Maryland; Johns Hopkins University School of Medicine, Baltimore, Maryland; Johns Hopkins University School of Medicine, Baltimore, Maryland; Johns Hopkins University School of Medicine, Baltimore, Maryland; Johns Hopkins Bayview Medical Center, Baltimore, Maryland; Johns Hopkins University School of Medicine, Baltimore, Maryland; Johns Hopkins University School of Medicine, Baltimore, Maryland; Johns Hopkins University School of Medicine, Baltimore, Maryland; Johns Hopkins University School of Medicine, Baltimore, Maryland; Johns Hopkins University School of Medicine, Baltimore, Maryland; Johns Hopkins University School of Medicine, Baltimore, Maryland; Johns Hopkins University School of Medicine, Baltimore, Maryland; Johns Hopkins Bayview Medical Center, Baltimore, Maryland; Johns Hopkins University School of Medicine, Baltimore, Maryland; Johns Hopkins University School of Medicine, Baltimore, Maryland; Johns Hopkins University School of Medicine, Baltimore, Maryland; Johns Hopkins University School of Medicine, Baltimore, Maryland; Johns Hopkins University School of Medicine, Baltimore, Maryland; Johns Hopkins University School of Medicine, Baltimore, Maryland; Johns Hopkins University School of Medicine, Baltimore, Maryland; Johns Hopkins Bayview Medical Center, Baltimore, Maryland; Johns Hopkins University School of Medicine, Baltimore, Maryland; Johns Hopkins University School of Medicine, Baltimore, Maryland; Johns Hopkins University School of Medicine, Baltimore, Maryland

## Abstract

**Introduction:**

The COVID-19 pandemic’s impact on school closures, workforce/employment status, and the enactment of stay-at-home orders may have resulted in changes in the incidence, mechanisms, and time to presentation for treatment of burn injuries. Given the need for prompt treatment of burn injuries to prevent worsening of the injury, infection, and/or critical illness, potential delays in burn treatment during the pandemic may have resulted in poorer patient outcomes. We hypothesize that burn patients demonstrated delays to presentation, changes in burn injury mechanisms, and increased mortality intra-pandemic compared to pre-pandemic.

**Methods:**

We conducted a retrospective review of records from the burn registry of a tertiary urban burn center. Persons aged 0 to 99 presenting with a burn between March 2019 and March 2021 were included for evaluation (n=1524). Demographics, time to presentation, and admission type were compared for persons presenting pre-pandemic (March 11, 2019-March 10, 2020) to intra-pandemic cases (March 11, 2020-March 10, 2021). TBSA, length of stay, mechanism of injury, and final disposition were compared for inpatients presenting pre- to intra-pandemic. Chi-squared and Student’s t-test analyses were conducted for comparisons between the pre- and intra-pandemic groups; statistical significance was set at p< 0.05.

**Results:**

Pre-pandemic, 777 persons presented to the burn center with 239 (30.8%) admitted; intra-pandemic, 747 persons presented with 260 (34.8%) admitted. Presenting patient mean age pre-pandemic was significantly lower than intra-pandemic (41 vs. 44 years, p=0.001), as well as mean time to admission (1.41 vs 2.46 days, p=0.04). There was no statistically significant difference in patient gender, race, ethnicity, or admission type pre- vs. intra-pandemic. For inpatient admissions (n=499), there was a significant increase in length of stay pre- vs. intra-pandemic (8.31 vs. 11.26 days, p=0.042); no significant changes in TBSA, mechanisms of burn injury, or disposition were found.

**Conclusions:**

Our results indicate that patients who presented intra-pandemic were significantly older and had greater injury-to-presentation times than those who presented pre-pandemic. The pandemic did not seem to have a significant effect on burn TBSA, mechanisms of injury, or disposition. These results may have important implications for the role of telehealth in burn care and public health programs for burn prevention and treatment during times of stress on the healthcare system.

**Applicability of Research to Practice:**

Analysis of pre- and intra-pandemic burn injuries enables explorations of how pandemics impact both the ability of patients to seek treatment and the impact of delays in seeking treatment. Such analysis lends itself well to exploring the vital role of public health advocacy, use of telehealth, and burn prevention campaigns during crises.

